# Manipulation under anesthesia for adult scoliosis pain: case series with 1-year follow-up

**DOI:** 10.1186/1748-7161-8-S2-P1

**Published:** 2013-09-18

**Authors:** Mark Morningstar

**Affiliations:** 1 Natural Wellness & Pain Relief Center, Richmond, MI, USA

## Background

Manipulation under anesthesia has been used in the U.S. since the 1920s, when it was primarily developed for the treatment of chronic or recurrent back and leg pain associated with intervertebral disc herniation or sciatica. 

## Purpose

The goal of this study was to report the radiographic and self-rated outcomes of adult scoliosis patients treated with manipulation under anesthesia (MUA). These patients had failed or plateaued with other non-surgical therapies. 

## Methods

A review of patient files who received MUA between January 2007 and March 2010 at the same outpatient surgery center. Of these, 17 patients had a history of adult scoliosis and had previously tried other conservative therapies for pain for at least six weeks. Patients ranged in age from 37 to 61 years. After reviewing the charts of these 17 patients, 14 of them had completed a 1-year follow-up. Outcome assessments included a Quadruple Visual Analog Scale (QVAS), Functional Rating Index (FRI) and Cobb angle. 

## Results

All 14 patients reported statistically significant outcomes on both the QVAS and the FRI immediately following the serial procedure, which were maintained at one year. The Cobb angle was reduced by an average of 11° in the lumbar group, 6° in the thoracic group and 4°/5° in the double major group. 
